# Novel Variable Radius Spiral–Shaped Micromixer: From Numerical Analysis to Experimental Validation

**DOI:** 10.3390/mi9110552

**Published:** 2018-10-27

**Authors:** Pouya Mehrdel, Shadi Karimi, Josep Farré-Lladós, Jasmina Casals-Terré

**Affiliations:** Mechanical Engineering Department—MicroTech Lab., Universitat Politècnica de Catalunya, Colom 7-11 08222 Terrassa, Barcelona, Spain; shadi.karimi@upc.edu (S.K.); josep.farre.llados@upc.edu (J.F.-L.); jasmina.casals@upc.edu (J.C.-T.)

**Keywords:** point-of-care, passive mixer, micromixer, spiral micromixer, mixing

## Abstract

A novel type of spiral micromixer with expansion and contraction parts is presented in order to enhance the mixing quality in the low Reynolds number regimes for point-of-care tests (POCT). Three classes of micromixers with different numbers of loops and modified geometries were studied. Numerical simulation was performed to study the flow behavior and mixing performance solving the steady-state Navier–Stokes and the convection-diffusion equations in the Reynolds range of 0.1–10.0. Comparisons between the mixers with and without expansion parts were made to illustrate the effect of disturbing the streamlines on the mixing performance. Image analysis of the mixing results from fabricated micromixers was used to verify the results of the simulations. Since the proposed mixer provides up to 92% of homogeneity at *Re* 1.0, generating 442 Pa of pressure drop, this mixer makes a suitable candidate for research in the POCT field.

## 1. Introduction

Microfluidics has opened new horizons in the biological fields [1], such as single cell study [2,3], drug discovery [4], lab-on-a-chip (LOC) and especially in point-of-care testing devices (POCT) development [4,5,6]. Microfluidics capabilities in flow cytometry has led researchers to conduct assays on evaluating the deformability of cells and permeability of drugs through certain membranes [7,8]. Besides, the controlled nature of the microfluidics has provided the possibility of sorting and sequencing of cells [9,10]. In recent years, development of point-of-care testing devices (POCT) by deployment of lab-on-a-chip (LOC) technology has been found to be noteworthy by the researchers [11]. Reducing the laboratory works and costs, rapid and accurate respond, providing bedside analysis and being user-friendly have been the most well-known characteristics of these devices [11,12]. Micromixers are one of the most important components of such devices. They have proved their efficiency in achieving objectives such as sample preparation, glucose concentration detection, blood plasma mixing, particle concentration detection etc. [13,14].

At high Reynolds number flows, mixing can be enhanced through secondary flows, chaotic advection and Dean vortices, but in microfluidics (laminar flows), mixing relies on diffusion. Therefore, according to the method used to maximize the mixing, micromixers can be classified as active or passive. Active micromixers require an external energy source to stimulate the perturbations in the flow, such as vibrations, and acoustic and electromagnetic stimulations. Passive micromixers do not require an external energy source and hinge on geometrical features of the design for providing a well-mixed mixture [13,14].

A lot of research has been conducted on optimizing the mixing phenomena in micromixers. Some researchers have engaged the features of both types of micromixers in one device. Afzal et al. [15] investigated the effect of combining pulsatile flow and a bulb-shape geometry on the efficiency of the mixer. In a similar approach, Silva et al. [16] carried out an investigation on a hybrid generation of micromixers, where variable inlet widths were coupled with the pulsed flow. Other researchers focused on the advantage of chaotic advection. For instance, Hermann et al. [17] studied the mixing in three types of split-and-recombine (SAR) micromixers. In the proposed mixers, the 3D structure of the micromixer provided the chance for the flow to split and twist and recombine with the initial flow from another side. Raza et al. [18] showed that a split-and-recombine micromixer with a 3D structure was able to provide high-quality mixtures at Reynolds numbers higher than 30. Chen et al. [19] compared a 3D split-and-recombine micromixer with an in-plane T-mixer and showed the improvement of this method against the simple in-plane mixer. Although hybrid and 3D generation of the mixers are able to provide mixtures with promising homogeneity, the complexity of fabrication and the increased pressure loss practically restricts the applicability of such devices in the development of lab-on-a-chip (LOC) devices and point-of-care tests (POCT).

In planar mixers, researchers mainly follow the increasing contact surface (for low Reynolds number regimes) or induce chaotic advection (for high Reynolds number regimes) to improve the mixing. Julios et al. [20] studied a planar mixer with perpendicular rectangular grooves on the straight microchannel. Alam et al. [21] also performed a similar investigation on the effects of adding rectangular grooves on a curved microchannel. Both studies provided acceptable results, especially on Reynolds number regimes higher than 60. Li et al. [22] studied the impact of putting obstacles on the path of the flow and showed that the placement, size and the angle of the obstacles can determine the performance of mixers in the range of 1 ≤ *Re* ≤ 10, but in higher flow rates the configuration of the obstacles does not play such an important role. Chen et al. [23] included a pattern of pillars in the flow path of the micromixer. The study clarified the relation between the Peclet number and the mixing efficiency. The microchannel with obstacles (higher Peclet number) translated into a higher efficiency mixer. Rahman Nezhad et al. [24] showed that deflecting flow with baffles could be a simple answer for acquiring relatively homogeneous mixtures, when the facilities are not advanced enough to fabricate sophisticated geometries. A study on the combination of gaps and baffles were conducted by Xia et al. [25]. The results showed that the micromixer was able to provide mixtures with significant uniformity at very low Reynolds number (*Re* < 1) and also at high Reynolds numbers (*Re* ≥ 40).

An alternative approach analyzed the effect of the geometry and working condition on the flow behavior. Hossain et al. [26] evaluated the mixing phenomena in three planar micromixers in a wide range of Reynolds numbers (0.267–250). This study showed that even though at the Reynolds numbers range of 20–100 the square wave channel provided the highest quality mixture compared to zig-zag and curved channels, at higher Reynolds numbers, the performance of the three mixers was almost identical. Khosravi Parsa et al. [27] investigated the mixing efficiency in a sinusoidal micromixer with respect to the ratio of amplitude to wavelength. The study concluded that at high Reynolds numbers, the geometry of the mixers with higher amplitude and smaller wavelength allowed the Dean vortices to develop in the peak zones. This was later translated into better mixing compared to the other models. Vatankhah [28] evaluated the effect of the channel’s cross-section dimensions on the velocity profiles in the channel. An accordion shape micromixer, which is an amended derivation of a zig-zag microchannel, was studied by Cosentino et al. [29]. Their study evaluated the characteristics of the proposed device, with respect to the biological applications and limits. The study indicated that not all the current mixers are suitable for biological and biomedical research, since they cannot provide sufficient mixing and support vital living conditions for cells. Researchers have addressed the problem of providing high quality mixtures at very high or very low Reynolds numbers. The low Reynolds number range (0.1 ≤ *Re* ≤ 10.0) is the Achilles ankle of micromixers and in all the references the lowest quality is reported in this domain. Moreover, the pressure drop has always been sacrificed in order to achieve a high quality mixture. However, an increased pressure drop might mean increased surface tension, which could damage or modify living cells.

The purpose of this paper is to introduce a micromixer suitable to be mounted on POCTs and LOCs. Since in these applications the Reynolds number is low, diffusion is considered as the main mixing mechanism, and the proposed design enhances the contact surface with a reduced pressure drop to provide tolerable conditions for living cells. The approach used in this study aims to enhance the residence time by enhancing the mixing phenomena using a novel planar spiral shape micromixer with expansion and contraction parts. The purpose of these expansions/contractions is to enhance the diffusion length and modify the velocity profile. Nine different mixers (one-loop, two-loop and three-loops without expansion, with 5% expansion and 10% expansion) have been used in this study to predict an improved performance in the critical working range of Reynolds numbers 0.1 to 10.0.

## 2. Mixer Design and Numerical Analysis 

### 2.1. Target Models and the Novel Modification

**Figure 1:**
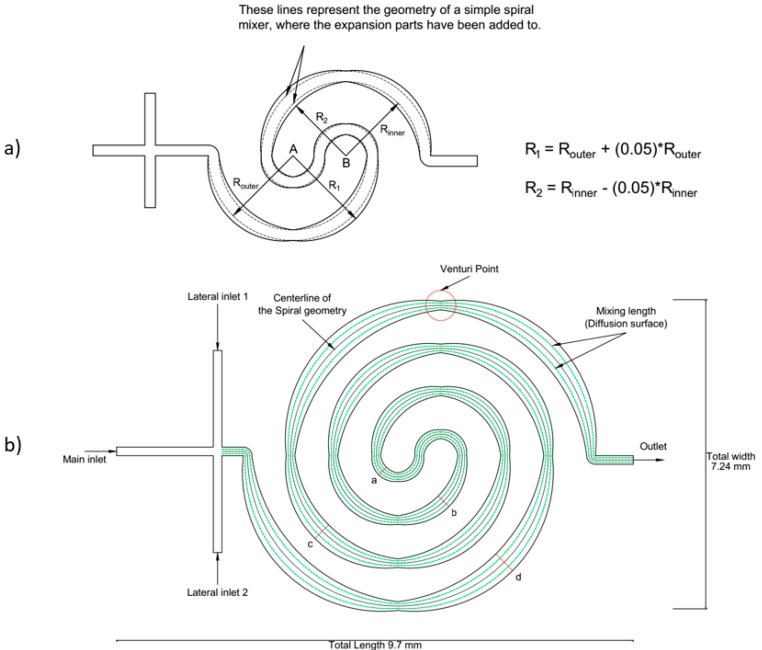
(**a**) Conceptual diagram of the proposed one loop class micromixer, where Rinner and Router represent the radius of inner and outer walls of the basic design of the spiral mixer. (**b**) Schematic view of the three loops mixer with 5% expansion. The width of the channel at the cross-sections a, b, c and d is 250, 350, 450 and 550 μm, respectively.

Three different classes of mixers will be studied based on a single loop, two loops and three loops. The successive arc-shaped mixing units are created from two different centers: A and B, see Figure 1a.

Expansions will be added to all three mixer classes from the simple spiral design, see Figure 1a (dash line). The initial type is the simple spiral micromixer without expansion (0%). Two more types of expansion/contraction mixers will be studied (5% and 10%). To build this geometry, the outer/inner wall of the microchannel is increased/decreased following a different circle path. These circles have a radius of 5% or 10% (based on the type of the mixer) difference from the original radius. The expansion parts are assumed to reach to maximum values at each arc at the π/4 and 3π/4 or 5π/4 and 7π/4 (depending on where the curvature is located). In all cases, a minimum width of 200 μm is achieved at nπ/2 points (Venturi point, see Figure 1b).

Shortly after the cross-shaped inlet, the centerline of the microchannel enters in an arc with an angle of 180° around the center point A. Then it continues its path with the same angle, but this time around center point B. This process continues until the arc reaches the exact midpoint between the centerlines A and B. After that, the radius of the arc increases, as the end of the arc recedes from the other center point. This process will go on until the outgoing curvature, which ends at the outlet, reaches the same value of the radius as the ingoing curvature. Table 1 summarizes the overall dimensions of the studied model and the total mixing length.

The proposed mixer, shown in Figure 1, minimizes the space consumption and does not increase the pressure drop as much as some types of micromixers such as split and recombine mixers and mixers with obstacles on the path of the fluid [17,18,22,23].

Instead of adding commonly used T or Y inlets, a cross-shaped inlet is used in the proposed mixer design. Therefore, a double interfacial area, as depicted in Figure 1b, is achieved by injecting the solvent symmetrically from two sides. The width of the channel in the inlet part is kept constant at 200 μm. The successive arrangement of arc-shape mixing units causes continuous acceleration and deceleration of the fluid flow, i.e., disturbing streamlines and enhancing diffusion.

### 2.2. Numerical Simulation, Governing Equations and Dimensionless Numbers

Fluid flow in micromixers is generally isothermal, incompressible, Newtonian and laminar. Therefore, it is governed by the continuity equation (Equation (1)), and Momentum (Navier–Stokes) equation (Equation (2)). Convection-Diffusion equation (Equation (3)) is the main physical phenomenon governing the mixing in such systems. In order to calculate the mixing, Equation (3) should be solved together with Equations (1) and (2), so the spreading concentration and the velocity field are coupled. These equations can be expressed, respectively, as:

(1) $$\nabla \vec{U}=0\;,$$

(2) $$\;\rho \vec{U}\cdot \;\nabla \vec{U}=-\nabla P+\;\mu \nabla^{2}\vec{U\;},$$

(3) $$\rho \vec{U}\cdot \nabla \varphi =D\nabla^{2}\varphi \;.$$

In the above equations, $\vec{U}$ is the velocity vector, ρ is the density, P is the pressure, μ is the dynamic viscosity, D is the diffusion coefficient and the φ is the concentration of the species inside the micromixer.

Numerous investigations have established the working conditions of micromixers according to the Reynolds number. This dimensionless number expresses the ratio of the magnitude of the inertial term to the viscous term in the channel.

(4) $$Re=\frac{\rho \vec{U}D_{h}}{\mu }\;,$$

where ρ, $\vec{U}$, and μ are density, velocity vector and the dynamic viscosity of the fluid, respectively, and the $D_{h}$ is the hydraulic diameter of the channel, for which a rectangular duct can be represented as:

(5) $$D_{h}=\frac{4\times A_{c}}{P_{w}}\;,$$

where $A_{c}$ is the surface area of the channel’s cross-section, and $P_{w}$ is the wetted perimeter.

Since the fluid flow in POCT micromixers is within the laminar range, the mixing can therefore be carried out via diffusion and/or advection. Depending on the operating Reynolds number, one of the above-mentioned effects is most likely to be overcome as the leading mixing factor in the device (except for the Re=1, where neither diffusion nor advection has dominance over the other). Peclet number (Pe) denotes the ratio of the advective transport rate to the diffusive transport rate.

(6) $$Pe=\frac{L\times \vec{U}}{D},$$

where L is the characteristic length.

In order to verify the validity of proposed design, the normalized ratio (σ) of the differences of the mixture and the mixing species to the ideal concentration is measured by analyzing the species distribution along the cross-section at the outlet of the mixer.

(7) $$\sigma =\frac{C_{i}-\;\bar{C}}{C_{\max}-\;\bar{C}}\;,$$

In the above equation, $C_{i}$ stands for the concentration at each pixel (position on the cross-section of the outlet) and $C_{\max}$ is the highest value of concentration in the mixer. Notably, $\bar{C}$ (ideal concentration) represents the median value between the maximum amount and minimum amount of concentration in the mixer, which in this study is set to 0.025 mol/m^3^.

(8) $$M\times Q=1-\sqrt{\frac{1}{N}\times \;\sum_{1}^{N}\left(\sigma^{2}\right)\;},$$

Mixing quality (M.Q) can be obtained through the abovementioned equation, where N is the number of calculating points (evaluating points) over the outlet.

The mixing process is simulated by a commercial CFD-code software, ANSYS FLUENT 15.0 (ANSYS, Inc., Canonsburg, PA, USA), which analyzes the flow and the diffusion in the device. Benefiting from Finite Volume approach, this solver solves the continuity and the momentum equations, at steady-state case, at each control volume with respect to the physical properties of the introduced fluids (such as density, viscosity and diffusivity), initial settings (pressure-induced or given velocity) and boundary conditions (see Table 2 for details about the physical properties). The solver is set to the SIMPLE scheme for Pressure-Velocity coupling. The configuration of the software for solving pressure and momentum modules is set to the second order method and for the scalar to the power law method. The software is capable of introducing scalars as non-reacting agents that do not change the density or the viscosity of the working fluid. The only distinguishing factor of the scalars from the dominant fluid is the different diffusion coefficient, which can be set in the software. The flow is assumed to be in the laminar range (*Re* smaller than 50) and therefore, Dean vortices are unlikely to take place. The middle inlet is dedicated to water and the diffusive agents are considered to be injected from the side inlets. The accumulative discharge of the side inlets is considered to be the same as the middle inlet.

**Figure 2:**
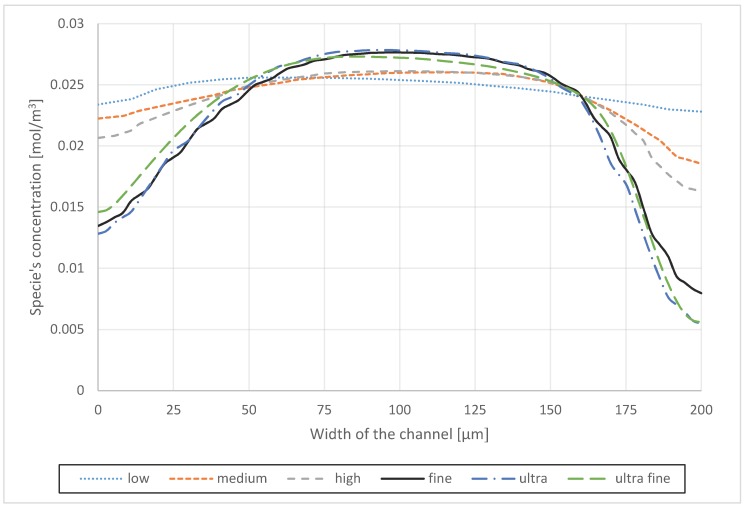
Distribution of the species concentration over a cross-section at the outlet of a simple one loop spiral micro mixer obtained from different meshes.

The 2D geometry is meshed in GAMBIT 2.4.6 software (ANSYS, Inc.) (the device’s dimensions are mentioned in Table 1). Therefore, a quadratic mesh scheme followed by a more detailed mesh distribution on the walls and critical points (Venturi points) is adopted. A grid independency analysis is done, based on six different mesh densities and setting the residuals to 10^−6^. Based on the meshing categories, the number of grid cells varied from 40,000 cells as the low-quality scheme for the one loop model without expansion parts to more than 3.1 million cells as the ultra-fine scheme for the three loops mixer with 10% expansion parts. However, by adopting the fine scheme, these ranges were limited to 289,000 and 1.7 million grid cells. The mean interval size of each cell varied from 10 μm to 2.75 μm (see Table 3). Among those, the meshing configuration that provided 3.75 μm of the mean interval size of each cell resulted in an acceptable answer accuracy (see Figure 2). According to grid independency analysis results, the mesh with cells smaller than 2.75 μm showed no significant change on the results but the required calculation time increased exponentially, see Figure 2.

## 3. Experimental Section and Fabrication Process

### 3.1. Fabrication of the Micromixer

Microfluidic mixers can be manufactured following different processes, varying from CO_2_ laser ablation to 3D printing techniques. The present investigation uses the soft lithography technique [31]. In this method, a cover glass slide is coated with photo-resist SU-8 (thickness 5-27 μm @GERSTELTEC SARL, Pully, Switzerland) to create a mold with the height of 25 μm. By placing a negative film of the micromixer and beaming UV light on it, the geometry of the device is defined in SU8. The mold fabrication process finishes with sinking the defined pattern in the SU8 developer (PGMEA—Propylene glycol methyl ether acetate @GERSTELTEC SARL, Pully, Switzerland) and cleaning it with propanol. Then, a 10:1 mixture of Silicone Elastomer SYLGARD 184 (@Dow corning, Midland, MI, USA) and its curing agent are poured on the mold, which previously is coated with chlorotrimethylsilane vapor. After baking the polydimethylsiloxane (PDMS) and removing the PDMS from the mold, the connections are created. O_2_ Plasma (@Gambetti Vacuum Technology, Binasco (Milan), Italy) is used to bond the hardened PDMS and a cover glass slide. Further precise measurements showed that in some mixers a maximum fabrication error of 2 μm in the width of the channel and 1.5 μm in the height of the channel was occurred. 

### 3.2. Experimental Setup

To generate the flow, two syringe pumps (Graseby 3200- @Smiths, Watford, Hertfordshire, UK- and kdScientific 410-CE- @kdScientific, Holliston, MA, USA) were used. Deionized water and food colorant (a solute of deionized water and food coloring -E133- with the ratio of 2:1) were used as the working fluids. According to the analysis, actual differences of the density and viscosity of the working fluids were negligible. The syringe pumps’ calibration was verified by filling a 5-mL container in a determined time, where the container’s volume was graded by 0.1-mL lines. 

The images of the unmixed and mixed fluids were captured using a Leica microscope (LEICA EZ4D- @Leica, Wetzlar, Germany), with 30× magnification. The microscope was equipped with lights that are able to beam on the top, side and the bottom of the device. The software of the microscope renders the image, varying from black and white view (0% of color saturation), normal view (100% of saturation) and exaggerated view (200% of saturation).

### 3.3. Image Analysis

Analysis based on images has been widely used in science. It is one of the most common methods to visually illustrate the results of the study. This method has a wide application varying from particle locating and tracking [32], to showing the lysis of cells and to the evaluation of the mixture quality [5]. 

In this study, pictures obtained of the mixing process are evaluated using ImageJ 1.51k software (National Institutes of Health (NIH), Bethesda, MD, USA). This software analyzes the features of a specified zone or line. In the evaluation process, the pictures are converted to grey scale (through RGB (Red, Green and Blue lights) analysis) and each pixel intensity is evaluated between 0 and 255. To reduce the noise influence, multiple analyses of the same zone from different pictures are carried out, and an average value is obtained as the foundation of the results. Notably, the standard deviation of the analysis is considered as the error range at each pixel.

Furthermore, experimental uncertainty in this work is highly influenced by the image processing section rather than gripping to the established boundary conditions. The results of image analysis showed that the intensity of light in the background varies from center to the corners and in the places where the light intensity was relatively lower compare to the center, higher noise was recorded in the RGB analysis. Background noise due to polarization and light breakdown, when it passed through the cover glass, PDMS layer and microscope lens, was obvious. For tackling this problem, it was decided to manually define a circular zone in the center of the photo capturing area in order to make sure that the minimum and maximum values of grey scale index are the same in the circle for all the experiments (Dettmer et al. [33] followed a similar process with software for reducing the background noise). This procedure proved to be useful as the reported noise dropped significantly.

## 4. Results and Discussion

### 4.1. Simulation Results Based on the Number of Loops

To investigate the influence of expansion in the mixing behavior, a basic spiral mixer was chosen to compare with mixers with different expansion rates up to 10% expansion. Numerical simulations were performed in a range of Reynolds numbers relevant to point-of-care microfluidic devices (0.1 ≤ Re ≤10.0). In the range of Re ≤0.9, molecular diffusion is the main mixing factor as discussed in methodology Section 2.2. The other two Reynolds regimes (*Re* ≈ 1 and *Re* ≤ 10) capture the balance between inertial and viscous terms when they are identical (*Re* = 1) and when the inertial term is dominating the mixing phenomena (*Re* = 10). 

Table 4 displays the general efficiency of the mixers calculated according to (Equation (8))**,** under Reynolds of 0.1, 1.0 and 10.0. The results show that the mixers can provide a mixture with a minimum quality of 62% and a maximum quality of 99.8%. Notably, the minimum quality of the final mixture is reported at the range of *Re* 1.0.

**Figure 3:**
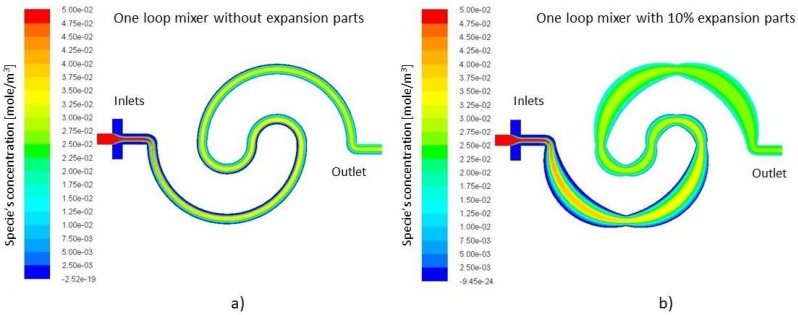
Simulation of the mixing of dyed water injected from inlet 1 and 2 and deionized water from inlet 3 at the Reynolds number of 1.0 (**a**) in one loop mixer without expansion and (**b**) one loop mixer with 10% expansion. The scalar concentration is defined as 0.05 mol for fluid 1 and 0 for fluid 2. The perfect mixing would give a scalar concentration value of 0.025.

Figure 3 illustrates that by utilizing expansion and contraction, the spiral mixer will increase the mixing length (or in another word the diffusion surface, see Figure 1b dashed line) along the channel. The numerical simulation (Figure 3) shows that since the flow is laminar, the flow lines expand following the profile, and therefore the diffusion surface increases. Therefore, the channel walls’ curvature affects the neighboring flow streams. Owing to the tiny scale of the micromixer, any changes in the flow stream’s direction and magnitude should not be neglected. As it is illustrated in Table 4, the mixing length of the devices increases by adding expansion parts compared to the basic mixers. The calculated mixing lengths of the studied models increase by a minimum of 27.18%, 17.25% and 9.31% compared to their basic designs. This increased mixing length will improve the performance of the modified spirals compared to simple spiral micromixers, but this will not be the only determining factor in evaluating the mixers’ performance.

**Figure 4:**
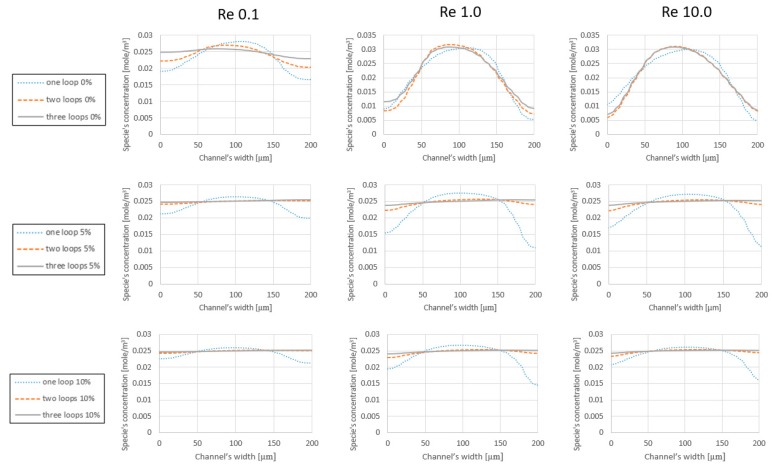
Species concentration at the outlet of the mixer. At each row, mixer types with similar expansion rates are compared with respect to the operating conditions, which are *Re* 0.1, *Re* 1.0 and *Re* 10.0. Regarding the definition of mixing, a straight horizontal line on the level of 0.025 (mol) represents an ideal prepared mixture at the outlet of the device.

In order to analyze the effect of increasing the number of loops and the added expansion parts on the mixing efficiency, the models are tested under similar initial conditions. Figure 4 displays the concentration of species along the cross-section line of the outlet (200 μm). At each graph, the expansion rate and the initial velocity are kept constant. According to the graphs, in the ranges of (Re<0.5) where the viscous term is stronger than the inertial term, the mixing is improved by adding loops. This can be translated into the fact that the species have more time to get diffused inside the channel and that the centrifugal forces are negligible. For the range of (Re=1.0), neither the viscous term nor the inertial term are dominating the flow. Therefore, although adding loops increases the length of the channel, the reinforced inertia of the flow does not allow the components to disperse into its surroundings, and even the added length is not sufficient to provide a significant improvement. This subject is repeated for the cases without expansion parts at (Re=10.0) with a difference. Even though the inertial term is 10 times stronger than the viscous term, it is not enough to cause secondary flows and Dean vortices, which are considered as the main mixing factors in a laminar regime that usually takes place at (Re > 40.0) [23].

Table 4 shows that the performance of the mixers at Reynolds 10.0 improves compared to Reynolds 1.0 range; however, this change is not appreciable in Figure 4. Regarding the simulation results (see Table 4), the performance of the simple spiral mixers (without expansion parts) is highly sensitive to the operating conditions. For instance, the efficiency of the mixers may vary from 96% (for 3 loops mixer without expansion parts at *Re* 0.1) to 62% (one loop mixer without expansion parts at *Re* 1.0). This sensitivity is reduced dramatically by adding expansion parts, and a more uniform distribution of the solute can be achieved at the outlet of the mixer in any operation range. In the spiral mixers without expansion parts, the mean difference between the highest quality and the lowest quality is around 20%, while in the one loop mixer with expansion parts, 12% difference is reported between the best and worst performance of the mixer. The difference is minor and around 2% or 1% for two loops and three loops mixers with expansion parts, respectively.

Besides the analysis on the Peclet number at the widest section of the mixers, where the channel reaches its maximum width, it was showed that the increase of the channel width the Peclet number decreases. Due to the definition of the Peclet number, this reduction can be translated into the fact that the diffusive term has been reinforced. In calculation of the Peclet number at the cross section, the characteristic length changes into hydraulic diameter. Due to the fact that the applied modifications on the geometry were in the XY plane and the flow rate was constant, the increase in the hydraulic diameter and the reduction in the flow velocity are not proportional. This description has been well illustrated in Table 5, where by adding 10% expansion parts the advective term has reduced 57% and 76% in one loop mixer and three loop mixers, respectively.

In the methodology, we already described that the initial velocities are set to generate specific Reynolds number regimes based on the minimum cross-section of the channel (Venturi Point). In the expansion and contraction mixers, the flow is driven into sections with larger cross-sections. Nonetheless, the regional Reynolds number reduces according to the definitions of Equations (4) and (5) in these areas. On the other hand, the architecture of the design and the existence of the Venturi points provide a continuous disturbance in the velocity profile. All these factors together will result in an enhanced and improved mixer that can operate in a wide range of Reynolds numbers regimes.

### 4.2. Simulation Results Based on the Expansion Rate

**Figure 5:**
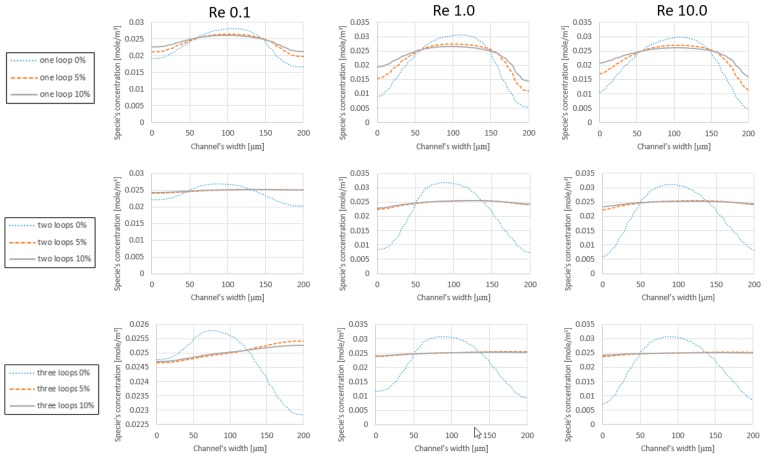
Species concentration along the outlet’s cross section. At each row, a specific class of the mixers is represented and at each column the concentration distribution of species at the outlets, regarding the operating conditions.

The mixer’s performance can be characterized from the expansion rate point of view. According to Figure 5, the one loop class of mixers with 5% and 10% expansion parts prepare the final solute with better quality. Although, regarding the analysis on the effect of the number of loops on the mixer’s efficiency, the performance of the different classes decreases in the *Re* 1.0 and *Re* 10.0 regimes. However, the mixer types with 5% and 10% expansion parts show significant improvement toward mixing efficiency. As displayed in all the graphs of Figure 5, the iconic revision in the design of the mixer damps the destructive effect of the inertial term in the mixing and reinforces the diffusion inside the device. Meanwhile, the quality of the one loop mixer without expansion parts is reported as 62% and 66% on *Re* 1.0 and *Re* 10.0 regimes, respectively. The performance of the one loop mixers with 5% and 10% expansion parts for the aforementioned regimes are reported as 85% and 88.8%. Moreover, the comparison between the mixer types in the two loops and three loops classes implies that adopting expansion and contraction features in the architecture of the mixer improve their efficiency by more than 30%.

### 4.3. Effect of the Expansion on the Diffusion

**Figure 6:**
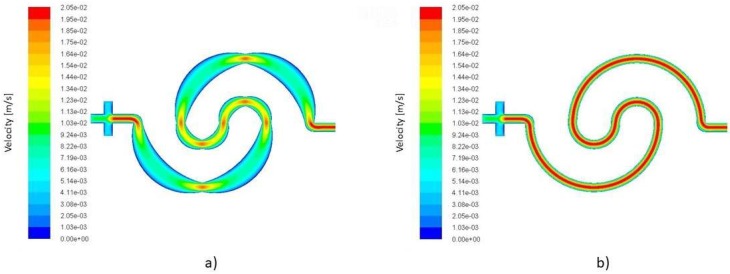
(**a**) Velocity contours of the one loop mixer with 10% expansion and (**b**) the one mixer without expansion parts. The operating condition for both mixers is identical (*Re* 1.0).

Adding loops to create as well as to add expansion parts to disturb the flow streams, increases the total mixing length. Both of these factors are key parameters in the Peclet number. Peclet number (Equation (6)) relies heavily on the mixing length and velocity vector; see Equation (6). Figure 6 shows the velocity contours of the one loop mixer without expansion parts and with 10% expansion parts. The distinguishing factor in the efficiency of the above mixers is the velocity profile and the extended mixing length. The combination of these parameters supports the mixing phenomena. The velocity is decreased and the mixing length is increased.

The global mixing efficiency at (Re=10.0) is improved toward mixers at (Re=1.0), but this trend changes completely in the mixers with expansion parts. According to the results, as Figure 5 illustrates, adding loops is an effective strategy for prompting the mixing length in the device. Although the device’s efficiency reduces at (Re=1.0) compared to other lower and higher initial velocities, the effect of the inertial term and the viscous term being in the same order has been weakened by adopting the expansion and contraction design. Due to the variable width of the channel and the changes in the flow direction at each segment, this geometrical feature causes a disturbance in flow stream and velocity profile continuously.

With respect to the continuity and momentum equations, the velocity at the Venturi points reaches the maximum value, and at the middle of each quarter arc, reduces to the minimum. This fluctuation can be repeated 8 to 16 times, depending on the type of mixer.

On the other hand, the simulation results indicate that the flow lines follow the curvature of the nearest wall, see Figure 6.

This means that the flow should cover more distance in the mixers with expansion parts compared to the models without expansion; see Table 6. Therefore, designs with expansion take advantage of an extended diffusion surface and longer diffusion time. Table 4 and Figure 5 clearly show that adding loops generates better mixing performance at all the mixing ranges. 

According to the results (see Table 4, Figure 4 and Figure 5), the quality of the final mixture that is prepared in a one loop micromixer with 5% expansion parts is improved by 33.71% compared to the one loop mixer without expansion parts. These improvements are reported as 88.89% and 94.3% for two loops and three loops mixers with 5% expansion parts, respectively, toward their simple models. Moreover, for devices with 10% expansion parts, these improvements advance further and peak at 52.25%, 93.6% and 96.9% for one loop, two loops and three loops mixers, respectively.

On the other hand, there is another important factor that plays a key role in the practicality and the efficiency of the proposed mixer. A numerous number of mixers are suffering from the considerable amounts of pressure drops in their designs. This feature causes the constant need for an external pump to overcome the pressure drop and keep the flow in a desired Reynolds number range. Besides increasing the chances of structural failure in the device, requiring an external energy source is always a drawback in the Point-of-Care research field. The investigations on the proposed micromixer show that adopting the expansion and contraction parts in the architecture of the design not only improves the mixing quality but reduces the pressure drop in the system. According to Table 7, the pressure loss at each class of the micromixers decreases by adding expansion parts. While the pressure drop for the one loop mixer without expansion parts is reported as 640.85 Pascal, that amount is reported as 442.48 Pa for three loops mixer with 10% expansion part (at Re=10.0). This can be translated into the fact that using the mixers with expansion parts can provide a higher mixing quality with a lower pressure drop.

### 4.4. Validating the Numerical Results Based on Real Case Experimental Models

Since the validity of the suggested idea should be verified, a series of experiments are designed and conducted to test the performance of the mixer. The devices from Table 8 (one loop without expansion parts, one loop with 10% expansion parts, three loops without expansion parts and three loops with 10% expansion parts) were fabricated and tested according to the simulated boundary and initial conditions. The constant flow rate of 0.2 mL/h was set for the main channel (water) and 0.1 mL/h was allocated to the side inlets (ink). Since the microscope beams the light from below and the dimensions of the channel are small, determining the borders of the channel is challenging. For a better contrast of the channel walls, the ink is injected from the lateral inlets. This allows a better identification of the channel walls and more accurate analysis of the device. Moreover, since the weakest performance of the micromixer was predicted to be in the *Re* 1.0 regime, the flow rate of the inlets was set to a value that mimics this regime.

**Figure 7:**
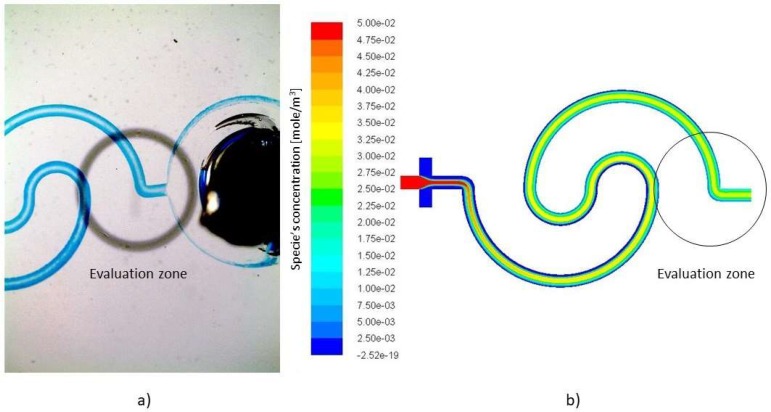
One loop simple spiral mixer: (**a**) Manufactured device species concentration, (**b**) Numerical simulation of the species concentration.

Figure 7 shows the image of the one loop mixer without expansion parts, which achieved a 58.28% mixing quality.

**Figure 8:**
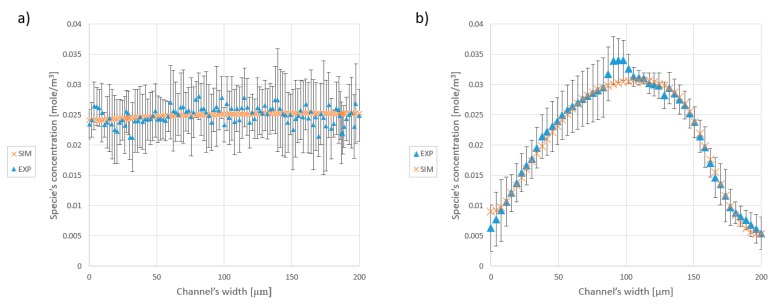
Concentration of the species at the cross-section of the outlet. (**a**) The graph represents the distribution of species at the outlet of the three loops mixer with 10% expansion. (**b**) Species distribution at the outlet of the one loop mixer without the expansion part.

Table 8 summarizes the comparison between simulation and experimental results. Several tests (at least three tests of each device and multiple pictures were captured during each test) provided the average standard deviation of the results, which can be translated into the error margin between the experimental and numerical results. The standard deviation increases in sample devices with larger geometries. This is due to the fact that to evaluate the mixture, the captured image should contain the inlets and the outlet. Therefore, the magnification, which is suitable for one loop mixer, is not adequate for the three loops class of spiral mixers. All in all, the average reported homogeneity of the fabricated mixers, obtained from pixel to pixel analysis, remains in a maximum 7% difference from the predicted numerical results. Thus, the numerical simulation and experimental results are in good agreement with experimental results. Figure 8 shows a comparison of the species concentration at the outlet (see evaluation zone in Figure 7). In this figure, the mean value of the greyscale at each pixel (analysis of multiple pictures of the evaluation zone) is considered as the result of the experimental evaluation and the error bars for each segment are determined through calculating the standard deviation of the analysis of the pictures in the related pixel. Due to the presence of noise in all the images, the calculations are carried out for the same spot from different pictures.

According to the results (Figure 8), not only is the global efficiency of the fabricated models in a reasonable range, but it also proves that the predictions about the concentration distribution, which were made by the software, are correct.

## 5. Conclusions

The purpose of this study was to design an improved micromixer for low Reynolds regimes useful for LOCs and POCTs devices with high efficiency and low pressure drop. The results of this paper propose a variable radius spiral micromixer as an excellent candidate. This paper studies the performance of the proposed designs in the flow regime range of Reynolds number 0.1 to 10.0. The numerical analysis was validated experimentally at *Re* 0.1.

Therefore, according to the results, the homogeneity of the mixture at the outlet is improved by adding loops to the mixer at low Reynolds numbers. By increasing the Reynolds number to 1.0, the efficiency of the micromixers without modified geometry dramatically drops, while in the variable radius micromixers, the decrease in performance at this flow regime is negligible. Both designs improved their performance at Reynolds 10. 

This work demonstrates that regardless of the number of loops, and at every given inlet flow, the performance of the variable radius micromixers improves by increasing the expansion rate. Combining a two loop or three loop micromixer with expansion and contraction parts achieves mixing efficiencies higher than 90%.

In addition, the proposed expansion and contraction parts decrease the pressure drop by more than 60%. This means that employing a two loop or three loop micromixer with expansion parts is a reliable choice in situations where it is required to ease the fabrication, provide a homogenous outcome, use the surface area efficiently and have a low pressure drop.

## Figures and Tables

**Table 1:** Overall dimensions of the studied models. The height of the channel at all sections is constant and set to 20 μm.

Name of the Device		Device’s Length (mm)	Device’s Width (mm)	Minimum Mixing Length (mm)
One loop	0% of expansion	5.7	3.2	14
	5% of expansion	5.7	3.23	14
	10% of expansion	5.7	3.26	14
Two loops	0% of expansion	7.7	5.2	29.7
	5% of expansion	7.7	5.22	29.7
	10% of expansion	7.7	5.3	29.7
Three loops	0% of expansion	9.7	7.2	51.7
	5% of expansion	9.7	7.24	51.7
	10% of expansion	9.7	9.36	51.7

**Table 2:** Physical properties of water and diluted ink.

Material	Density (kg/m^3^)	Viscosity (kg/(m·s))	Diffusivity in Water (m^2^/s)
Water	997	0.001	-
Diluted ink	≈997	≈0.001	5.5 × 10^−10^ [30]

**Table 3:** Meshing details of one loop micromixer with 0% of expansion.

Mesh Design	Interval Size of the Mesh	Minimum Orthogonal Quality	Maximum Aspect Ratio	Number of the Cells
Low	10	0.87	2.19	40,223
Medium	7.5	0.872	2.24	71,597
High	5	0.848	2.45	161,473
Fine	3.75	0.818	2.36	289,093
Ultra	3.25	0.83	2.51	383,679
Ultra-fine	2.75	0.839	2.38	537,233

**Table 4:** The efficiency of the mixers calculated according to Equation (8). The Reynolds numbers are based on the accumulative discharge of the inlets at the smallest cross-section of the channel.

Mixer Type		*Re* = 0.1	*Re* = 1.0	*Re* = 10.0
One loop	0% expansion	82.8%	62%	66%
	5% expansion	90.25%	77.6%	81.1%
	10% expansion	93.3%	85%	88.8%
Two loops	0% expansion	90.3%	61.2%	63.3%
	5% expansion	98.3%	96%	96.3%
	10% expansion	98.6%	96.9%	97.7%
Three loops	0% expansion	96%	67%	64.9%
	5% expansion	98.9%	97.9%	98.1%
	10% expansion	99.2%	98.5%	98.9%

**Table 5:** Peclet number at the widest cross section of the channel (*Re* 1.0).

Mixer Class	0% Expansion	5% Expansion	10% Expansion
One loop	1836	1092	777
Two loops	1836	859	561
Three loops	1836	708	439

**Table 6:** Effect of adding expansion parts on the increase of mixing length in comparison to the mixers without expansion parts.

Mixer Type		ΔL (mm)
One loop	0% expansion	-
	5% expansion	1.71
	10% expansion	1.73
Two loops	0% expansion	-
	5% expansion	2.30
	10% expansion	2.35
Three loops	0% expansion	-
	5% expansion	4.50
	10% expansion	4.63

**Table 7:** Pressure drop amongst various types of mixers. All the values are in Pascal.

Mixer Type		ΔP at *Re* 0.1	ΔP at *Re* 1.0	ΔP at *Re* 10.0
One loop	0% expansion	6.17	61.97	640.85
	5% expansion	3.26	32.78	352.9
	10% expansion	2.44	24.58	275.18
Two loops	0% expansion	12.6	126.23	1284.21
	5% expansion	4.78	47.96	506.84
	10% expansion	3.24	32.59	359.29
Three loops	0%expansion	21.61	216.3	2181.24
	5% expansion	6.03	63.48	664.153
	10% expansion	4.03	40.48	442.48

**Table 8:** Comparison of numerical and experimental mixer’s efficiency at *Re* 1.

Mixer Type		Simulation Results (*Re* 1.0)	Experimental Results
One loop	0% expansion	62%	58.2% ± 8.57%
	10% expansion	85%	78.9% ± 9.13%
Three loops	0% expansion	68%	66.3% ± 13.11%
	10% expansion	98.5%	91.8% ± 14.74%
